# Sex‐specific differences in pain localization in female patients with endometriosis: A comparison of sexless and female human body outlines

**DOI:** 10.1002/brb3.3285

**Published:** 2023-10-18

**Authors:** Natasza Szczypien, Zoe Ruchay, Zino Ruchay, Sandra Verena Müller, Claudia Kaiser, Frank Klawonn

**Affiliations:** ^1^ Institute for Information Engineering, Faculty of Computer Science Ostfalia University of Applied Sciences Wolfenbüttel Germany; ^2^ Faculty of Social Work Ostfalia University of Applied Sciences Wolfenbüttel Germany; ^3^ Clinic for Gynecology and Obstetrics University Hospital Schleswig‐Holstein (UKSH) Kiel Germany; ^4^ Biostatistics Group, Helmholtz Centre for Infection Research Braunschweig Germany

**Keywords:** endometriosis, pain drawing, pain profile, sex/gender differences

## Abstract

**Background:**

This study explores sex‐specific differences in pain localization using pain drawings in female patients with endometriosis. Traditional human body outlines (HBOs) used for pain drawings are often viewed as male, making accurate pain assessment difficult. The study aims to compare pain localization and extent between patients presented with sexless and female HBOs.

**Methods:**

A total of 49 female patients with endometriosis completed questionnaires and pain drawings (*n* = 24 and *n* = 26 with individually designed sexless and female HBOs, respectively). The Ruzika similarity index was used to investigate potential differences in pain drawings between sexless and female HBOs. Hypothesis testing was applied to compare the number of pixels marked in the pain extents and to investigate the suitability of the presented body outline.

**Results:**

Sex of HBOs used in pain drawings had no effect on pain area, and no statistically significant differences were found in pain localization or area between female and sexless outlines. Most, but not all participants found the body outlines suitable.

**Conclusions:**

The findings suggest that differences in the resulting areas marked in the pain drawings were negligible and the preferences for sexless pain drawings were not significant, so that a sexless body outline for pain drawings could be a good choice, especially when a study does not focus on one specific sex.

## Graphical abstract

This study investigated the influence of female versus gender‐neutral human body outlines used in pain drawings to localize pain caused by endometriosis. No significant in size or localization of pain areas could be detected between the two different types of pain drawings.

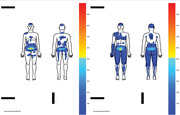

## 1 INTRODUCTION

Research has recognized sex differences in health and illness behaviors since the 1970s (Gassner et al., 2018). However, medical research has historically overlooked these differences. Until the 2000s, the male white body was considered the norm in medical research, diagnosis, and therapy, leading to the underuse, overuse, and misuse of both male and female populations (Gassner et al., 2018). Today, it is widely accepted that sex differences are relevant in all areas of healthcare, from prevention to treatment (Wattenberg et al., 2019). Recent studies have shown that sex differences play a significant role in the incidence of COVID‐19 (Brady et al., 2021) and in the immune response to vaccines and infections, with females showing a more effective immune response than males of the same age (Adland et al., 2020).

Pain drawings (PDs) are a commonly used tool in clinical practice to communicate subjective pain experiences (Barberro et al., 2015), and were first introduced by Palmer (1949). However, traditional human body outlines (HBOs) provided to patients are often viewed as androgynous but distinctly male (Cleeland & Ryan, 1994; Egsgaard et al., 2016; Türp et al., 1998), with few studies using sexless PDs (Barberro et al., 2015; Egsgaard et al., 2016; Jamison et al., 2011). Given that accurate pain assessment and communication is crucial for diagnosis and pain management, sex differences in pain sensation or perception should be taken into consideration (Chuk, 2002; Egsgaard et al., 2016). However, this requires HBOs that are inclusive for all individuals, including those who do not conform to the sex binary system (Gassner et al., 2018).

Studies have found that sex should be considered in pain drawings, with women drawing larger pain areas than men for chronic musculoskeletal pain (George et al., 2007). However, little attention has been given to increasing gender diversity in pain drawings and associated HBOs (Shaballout et al., 2019). Egsgaard et al., 2016 found that women did not show a preference for female or male PDs when drawing their pain, and even drew slightly smaller pain areas on male HBOs.

The aim of this study is to investigate sexless differences in pain localization using PDs in female patients with endometriosis who experience pain. The focus is not on the medical perspective of endometriosis pain, but on the PDs, pain profiles (PPs), and a questionnaire of endometriosis patients to examine whether there is a difference in pain localization and extent between those presented with sexless and female HBOs using PPs (Szczypien et al., n.d.). To calculate the statistics and measure the differences between these PPs, we developed a permutation test for our open‐source scientific software for pain drawing analysis Pain2D (https://www.pain2d.com/). Using questionnaires, the study also aimed to investigate how participants perceive and relate to the HBOs, and whether they view them as sexless or feminine.

## 2 METHODS

### 2.1 Procedure

**FIGURE 1:**
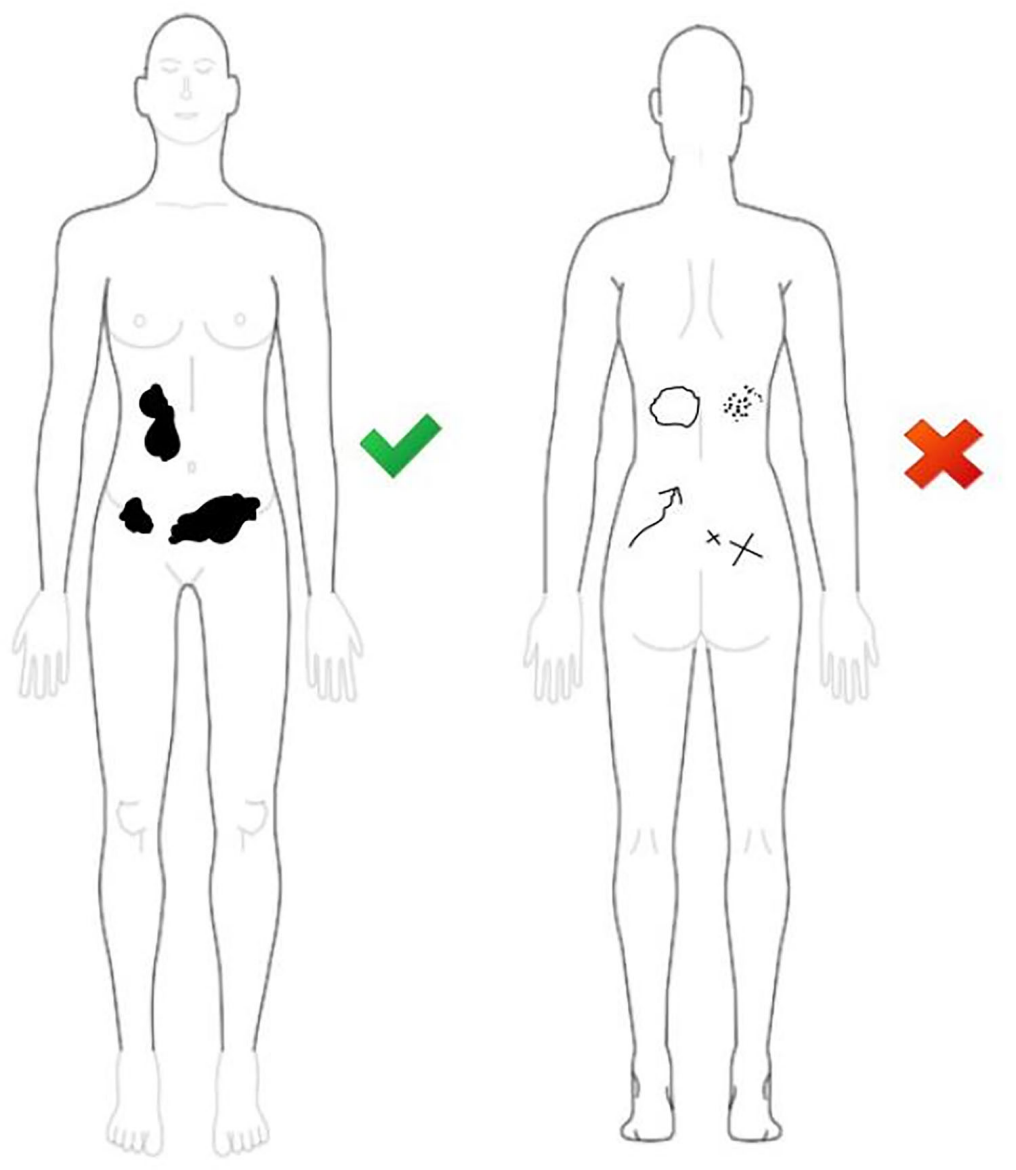
Drawing guide collected results for study patients showing correct (left) and incorrect (right) pain drawings.

Female patients who took part in the study were given a package containing five sheets in the waiting room. The first sheet provided a brief summary of the study and ensured that participation was voluntary and anonymous. The second sheet provided patients with guidelines on how to mark the areas of pain on a pen‐and‐paper template pain drawing (TPD) using either a sexless or female HBO created by Pain2D‐Designer (Szczypien et al., n.d.). Pain2D‐Designer is an easy‐to‐use tool for creating TPDs based on selection of HBOs. A detailed description can be found at http://www.pain2d.com/pain2d‐designer‐tutorial/index.html. The gray HBOs and three black indicators on the TPD allow a fast and efficient data export from paper sheets and for patients to mark their pain extents. Specifically, patients were instructed by a study sheet (see Supporting Information) to use a black fiber pen only to draw their endometriosis pain in the abdomen and back, including all the pain extents they felt. They were not allowed to use crosses, unfilled points, arrows, etc., as shown in the right part of Figure 1. Our approach differed from Barbero et al.’s (2015) study, in which a trained operator provided standardized verbal explanations on how to complete a PD on the tablet, to avoid confirmation bias (Nickerson, 1998).

**FIGURE 2:**
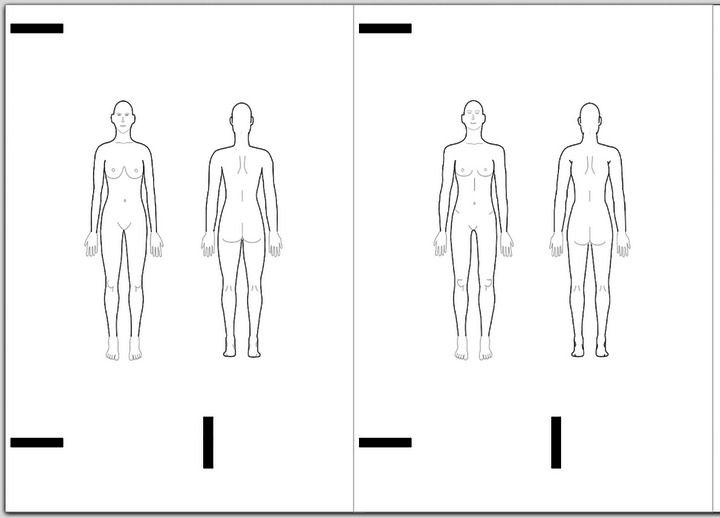
Template pain drawings generated by Pain2D‐Designer with individually created female (left) and sexless (right) human body outlines.

The third sheet was the TPD itself (see Figure 2), while the fourth sheet was a questionnaire (see Section 2.3.1). The final sheet provided more detailed information about the study. Each patient was provided with a pen to fill out the documents. To maintain anonymity, the TPD and corresponding questionnaire in each sheet were anonymized beforehand with an abbreviation consisting of the letter F (which stands for Female) or N (which stands for non‐sex) and a consecutive number separated by an underscore (e.g., F_21 means Female PD, Patient number 21).

### 2.2 Participants

Our current study included female patients with endometriosis who visited the Clinic for Gynecology and Obstetrics (gynecology) at the University Hospital Schleswig‐Holstein (UKSH) between March 15, 2021 and May 31, 2021. UKSH has a specialized endometriosis center that provides care for patients from across the state of Schleswig‐Holstein (cf..

Prior to the study, an inquiry was made to the UKSH internal Ethics Committee to determine if an ethics vote was necessary. Our study was performed according to the Declaration of Helsinki and the ethics committee confirmed that the research project did not require an ethics vote since it did not interfere with the psychological or physical integrity of the participants, nor did it involve the use of bodily materials or data that could be attributed to a specific individual. In order to comply with data protection guidelines, all data were anonymized, preventing the identification of participating individuals and avoiding processing of “special categories of personal data” (Art. 9 para. Para. 1 DSGVO). As such, explicit declaration of consent from participants was not required (Art. 9 (2) DSGVO).

**FIGURE 3:**
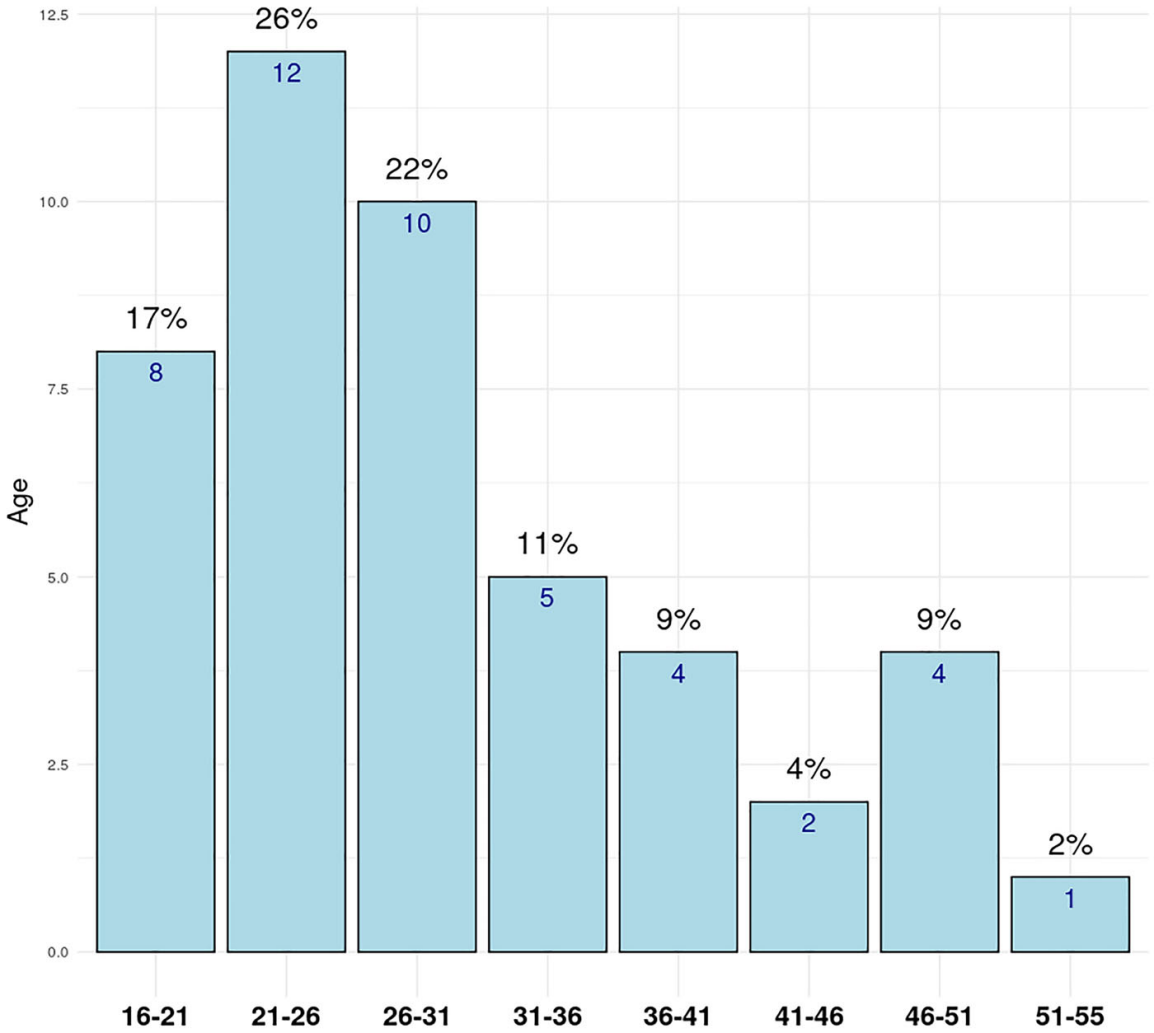
Age distribution of participants.

The study's participation was voluntary, and all patients were equally informed about the research project and conditions of participation. Consequently, a random sample of 49 female patients (*n* = 49; Age: mean = 30.2; median = 28; SD = 9.4, range = 16–55) with endometriosis completed questionnaires and pain drawings (PDs). Among the TPDs used, 24 had sexless (*n* = 24; Age: mean = 29.8; median = 28; SD = 8.7, range = 16–53) HBOs, and 26 had female (*n* = 26; Age: mean = 30.6; median = 28; SD = 10.2, range = 19–50) outlines. The median age is robust, accounting for unusually old or young subjects grouped by age. The majority of participants were between 21 and 25 years old, followed by the 26‐ to 30‐year‐old group (see Figure 3). Four subjects did not indicate their age.

### 2.3 Equipment

#### 2.3.1 Questionnaires

In order to investigate all hypotheses related to the HBOs presented in Table 2, we used EvaSys (2021) to construct and evaluate all collected questionnaires. For questions one and two (see Table 2), a binary scale was used, which reduces the effort required from both participants and researchers during assessment and evaluation, as suggested by Grassi et al. (2007). Despite some information loss compared to Likert scales, validity is not necessarily compromised. In fact, Awad et al. (2014) found that binary scales demonstrate higher inter‐rater reliability than Likert scales, and the simplification adds value. However, there is conflicting evidence in the literature regarding the reliability and validity of scales with less than five expressions, as Franzen (2019) points out. For questions four and five (see Table 2), a binary response scale with a neutral option was utilized, and we followed Porst's (2019) “10 commandments of question formulations.” The order of questions and responses was arranged to avoid any effects of question and answer order, such as halo (Jones & Reynolds, 2002) and response order effects (Robinson & Clore, 2002), as much as possible. Furthermore, we took survey artifacts like social desirability and demographic effects into account and avoided them to the best of our ability (Bogner & Landrock, 2015).

#### 2.3.2 Template pain drawings and body outlines

In this study we call a PD, a pen‐and‐paper pain drawing which consist of individual pain areas marked by patients with an Edding fiber pen 1200 black tip 1 mm. A TPD is a TPD which consists the female or sexless HBOs and does not contain any marked pain areas. Sexless and female TPDs with two views (frontal, dorsal) were prepared in an A4 paper with Pain2D‐Designer (Szczypien et al., n.d.).

For this study, we used the free software Inkscape (Inkscape, 2022) to design new female and sexless HBOs, using human anatomy as reference points (see Figure 2). We focused mainly on the breast, waist, hips, and shoulders, which are the main areas that show sex‐related differences (Egsgaard et al., 2016; Lippert et al., 2010). Feminine characteristics tend to include rounded and larger breasts and nipples, non‐muscular arms, wider and rounder legs and thighs, narrower waist, wider hips, and a rounder body shape, while masculine characteristics tend to include smaller breasts and nipples, broader shoulders, muscular arms, wider waist, narrower hips, and a straighter, more angular body shape (Frankl & Kriegel, 2019; Kelly et al., 2018; Lippert et al., 2010). On average, men have narrower hips and shoulders than women (in a biological sense) (Lippert et al., 2010; Schünke et al., 2014).

To create a sexless body outline, male and female anatomy information were combined. Slight adjustments were made to the waist and back to achieve higher neutrality. The gap between the thighs was slightly increased, and the breasts were changed in shape and size. Average nipple size was used for both sexes. The final outline appears sexless due to the width of the shoulders and the gap between the legs, as well as the attenuation of breast roundness.

To prevent any potential bias, the female HBO drawing area around the abdomen, hips, and waist was adjusted to match that of the sexless one, as endometriosis pain tends to occur more frequently in this area (Mechsner, 2016). This ensures that both outlines have the same size, preventing any distortions that may arise from perceived size differences. The larger the free areas, the larger the perceived pain extents, which could explain why women in Egsgaard et al.’s (2016) study drew slightly larger pain areas on female HBOs.

#### 2.3.3 Open‐source software Pain2D for pain drawing data collection and analysis

The Pain2D‐Designer software (Szczypien et al., n.d.) was used to generate the female and sexless TPDs. The HBOs were created individually (see Section 2.3.1) and imported into Pain2D‐Tool for easy pain point extraction and analysis including the deletion of possible marked pixels outside the HBOs. Pain2D‐Tool (Szczypien et al., n.d.) has four main functions: exporting marked pain extents as JSON files, generating PPs, validating disease classification models using a binary classifier and receiver operating characteristic (ROC) analysis area under the ROC curve (AUC), and generating statistics for disease separation using a k‐disease classifier (KDC) and nearest neighbor classifier.

#### 2.3.4 Permutation test (perm‐test) plugin developed for Pain2D‐Tool

**FIGURE 4:**
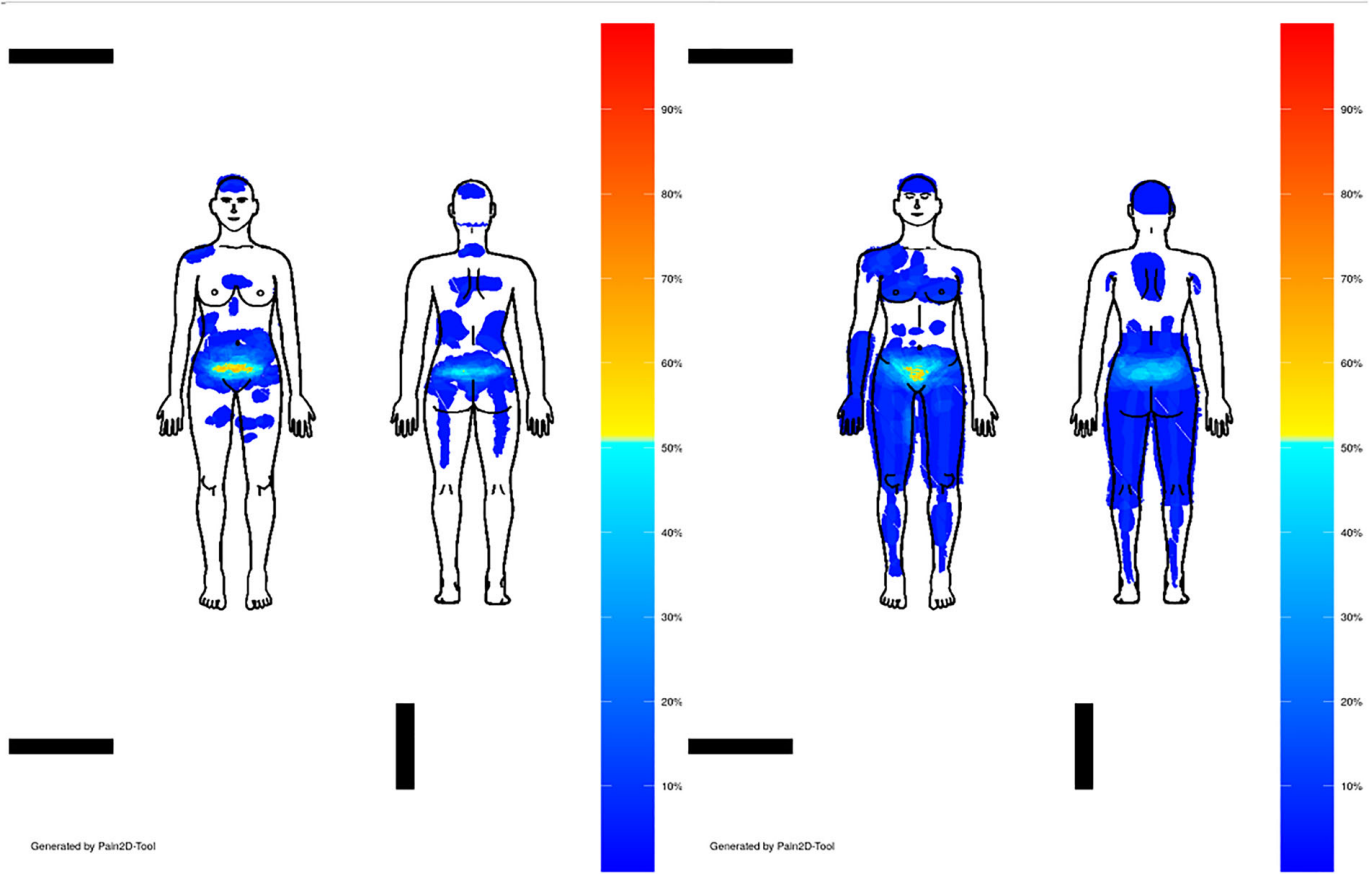
Female (left) and sexless (right) pain profile reveals characteristic pain clusters for endometriosis. The closer a value in the pain profile is to 1, the higher the proportion of persons in the corresponding group who have marked this pixel.

In this study, a permutation test plugin was developed for Pain2D‐Tool to investigate potential differences in PDs between two groups (e.g., sexes) within a disease. To conduct the permutation test, the two PD groups, for example, female and sexless were imported separately, and two PPs were created: for example, one PP for female and second for sexless PDs (see Figure 4).

To determine the differences between the two PD groups, the Ruzika similarity index (RZI) (Ružička, 1958) was calculated on both generated PPs (Origin RZI) on a scale of 0 (no similarity) to 1 (perfect similarity). The closer the RZI value is to 1, the more similar the two PPs are. RZI is an extension of the Jaccard index to non‐binary values, allowing us to compare also PPs that do not indicate whether a pixel was marked in a single PP but provide the proportion of PDs in a group that marked the corresponding pixel. For the comparison PPs and PDs a Jaccard index‐based, respectively, an RZI‐based approach seems to be more suitable than approaches that carry out a comparison on all pixels instead of focusing on only those pixels that were marked.

A permutation test was conducted to evaluate significant differences between the PDs of two groups. The groups were merged, and PD labels were shuffled while maintaining group proportions. RZIs were recalculated, and the process was repeated 1000 times to generate a null hypothesis distribution of RZI values. The *p*‐value was calculated by dividing the number of RZIs greater than the “Origin similarity” by the total number of permutation combinations, using a pre‐defined significance level alpha. This test determined whether any observed differences in PDs between two groups within a disease were statistically significant. A permutation distribution histogram is generated by the permutation test plugin including the null hypothesis RZI values, a *p*‐value, an “Origin similarity” and a preset alpha value.

### 2.4 Statistical analysis on pain drawings

#### 2.4.1 Comparing the differences between the pain extents drawn on female and sexless pain drawings (Hypothesis 1.1)

**FIGURE 5:**
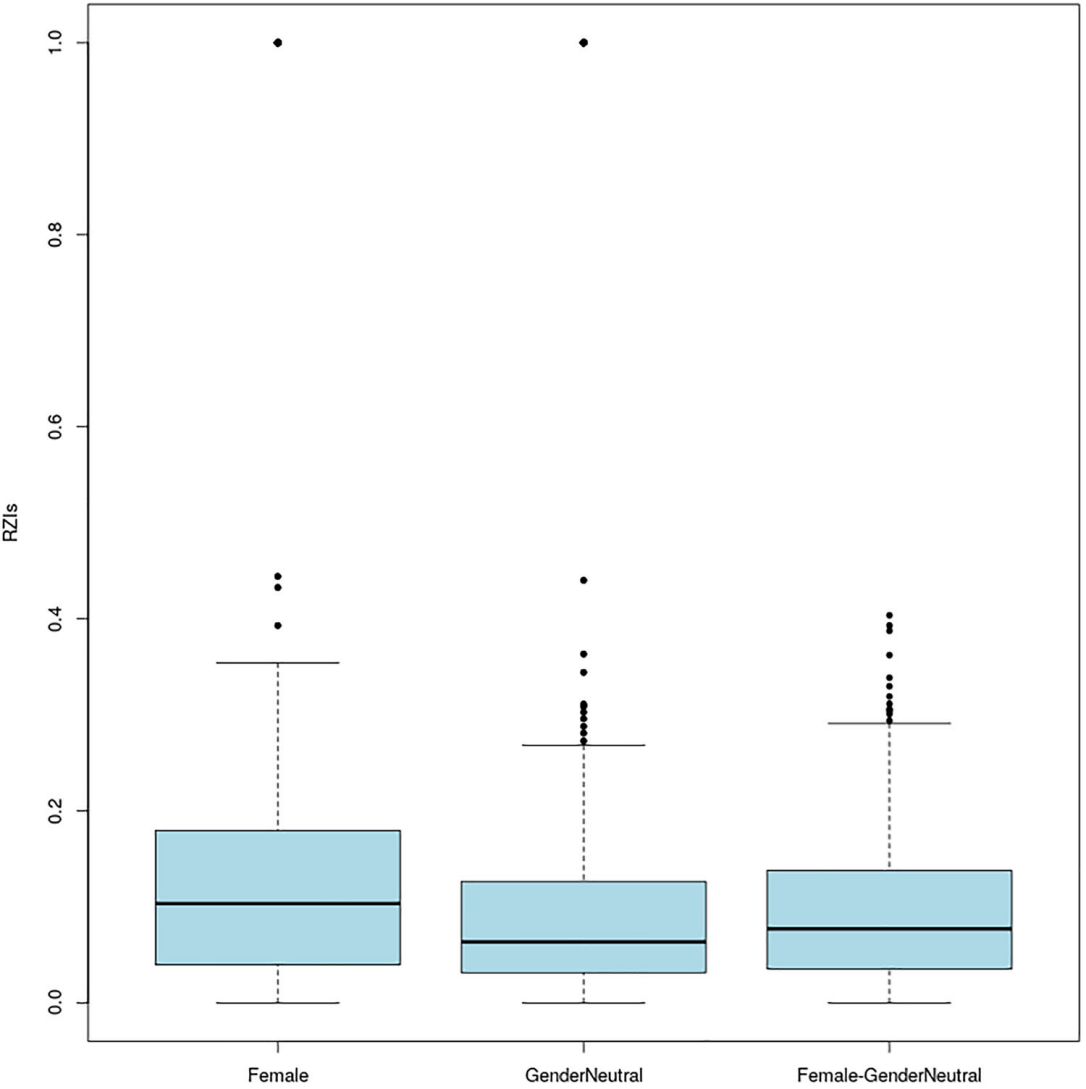
The boxplots summarize the calculated Ruzika similarity indices within the female, within the sexless and between the female and sexless pain drawing pairs.

**FIGURE 6:**
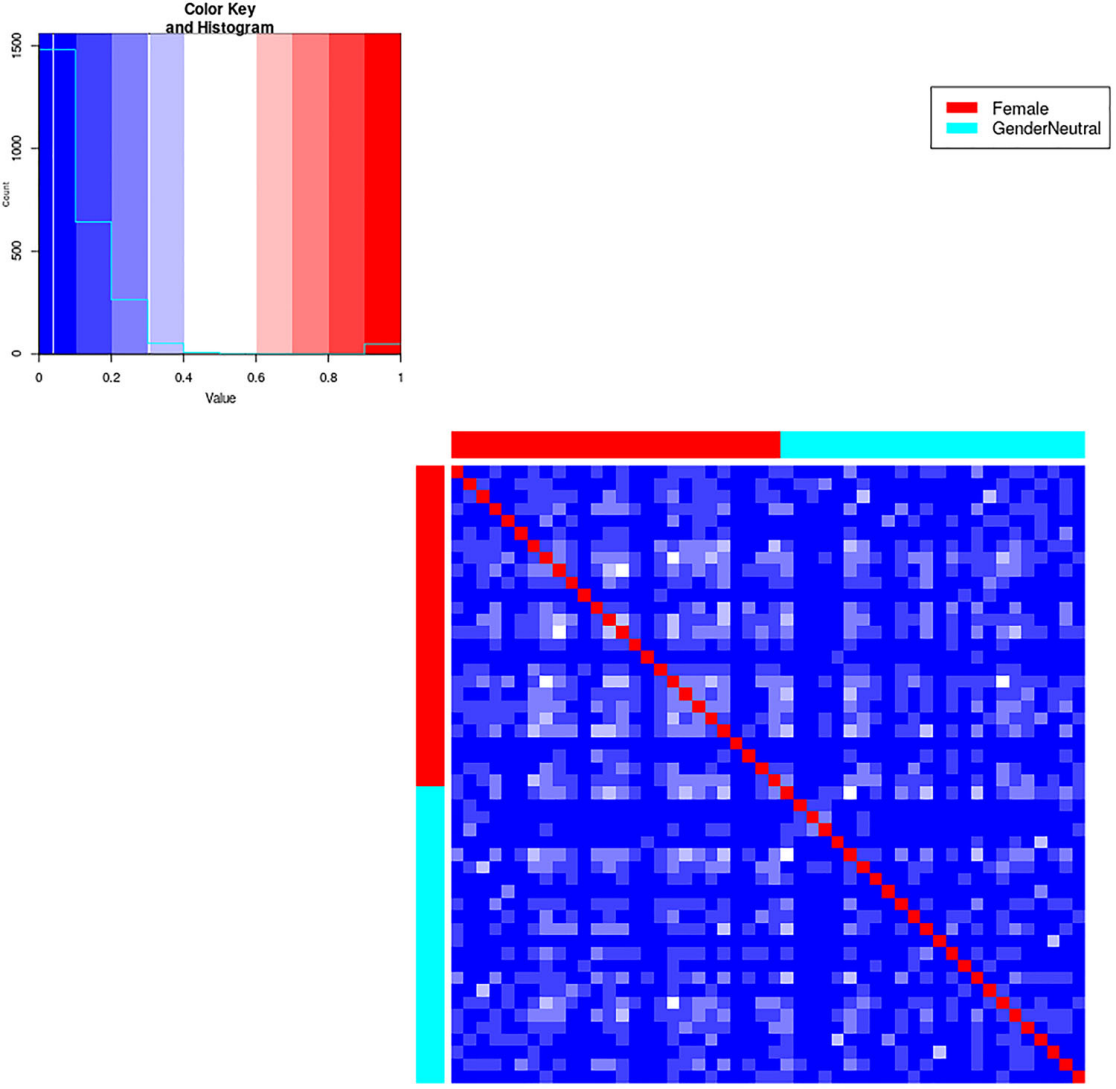
Heatmap generated by the nearest neighbor plugin in Pain2D‐Tool, which displays Ruzika similarity indices, with calculations performed only between pain drawings within the sexless (blue rows and columns) and female groups (red rows and columns), as shown in the upper left and lower right quadrants, respectively. The lower left and upper right quadrants are mirror images of each other along the diagonal. The sub‐squares in the heatmap do not show any visible differences in how pain extents are drawn within the female or sexless groups, or between these two groups.

In total, 49 pain drawings (PDs) were collected to determine whether there are statistically significant differences in endometriosis‐specific pain drawings between female and sexless HBOs. To achieve this we used the nearest neighbor plugin from Pain2D‐Tool (see Szczypien et al., tn.d.) which calculated RZIs between all PDs and displays them on a boxplot and heatmap (see Figures 5 and 6).

#### 2.4.2 Comparing the differences between the pain extents drawn on female and sexless pain profiles (Hypothesis 1.2)

Pain profiles were generated using Pain2D‐Tool, and a permutation test (described in Section 2.3.4) was performed on both female and sexless groups. For a pain profile (PP) of a group for each pixel the proportion of PDs in the group in which the corresponding pixel was marked is computed. In contrast to a PD, pixels do not have binary value in PPs but values between 0 and 1. The number of pixels marked on the pain diagrams for female and sexless patients was also compared to determine if endometriosis patients using female HBOs recorded their pain more comprehensively than those using sexless HBOs. For each group, the number of marked pixels in each pain diagram was counted, and the log10 of these values was subjected to both a Wilcoxon signed‐rank test and a two‐sample *t*‐test.

### 2.5 Statistical analysis on questionnaires

#### 2.5.1 Suitability of the HBOs (Hypothesis 2.1)

In order to assess the suitability of the designed HBOs for capturing endometriosis‐specific pain experienced by female patients, question 1 “Do you think the pain drawing is suitable for drawing pain on your body?” (see Table 1) with two possible responses (“yes” and “no”) was formulated. The responses were collected separately for the female and sexless groups, and Fisher's exact test was employed for analysis.

**TABLE 1:** This study used six questions along with their respective answers.

Nr.	Question	Possible answers
1	Do you think the pain drawing is suitable for drawing pain on your body?	Yes; No
2	Do you consider the following HBOs as sexless?	Yes; No
3	If yes, what characteristics do you use to see if the body outline is sexless or female?	Head; Shoulders; Arms; Legs; Buttocks; Abdomen; Waist; Chest; Feet
4	When you draw your endometriosis‐specific pain how important do you think it is that the HBOs is sex‐adapted?	Important; Unimportant; No opinion
5	How much does it bother you in the pain drawing if the body outline is not sex‐adapted?	Bothers me; Does not bother me; No opinion
6	Please indicate your age.	

#### 2.5.2 Sex neutrality or femininity of the HBOs (Hypothesis 2.2)

In order to detect the sex neutrality or femininity of the HBOs, question 2 (see Table 1) “Do you consider the following HBOs as sexless?” with two possible responses (“yes” and “no”) was formulated. The responses were collected separately for the female and sexless groups, and Fisher's exact test was employed for analysis.

#### 2.5.3 Sex recognition characteristics (Hypothesis 2.3)

In order to detect if there are specific features in the individually created HBOs that reveal the sex of the HBOs, question 3 (see Table 1) “If yes, what characteristics do you use to see if the body outline is sexless or female?” with categorical responses: Head; Shoulders; Arms; Legs; Buttocks; Abdomen; Waist; Chest; Feet were formulated. The responses were collected separately for the female and sexless groups, and a chi‐squared test was employed for analysis.

#### 2.5.4 Importance of identification (Hypothesis 2.4)

In order to detect if the identification with the body outline is important to endometriosis patients, question 4 (see Table 1) “When you draw in your endometriosis‐specific pain how important do you think it is that the HBO's sex is adapted?” with three possible responses (“important”; “unimportant”; and “no opinion”) were formulated. The responses were collected separately for the female and sexless groups, and Fisher's exact test was employed for analysis, dropping the “no opinion” answers.

#### 2.5.5 Perception of disturbance in case of non‐conformity (Hypothesis 2.5)

In order to detect if the identification with the body outline is important to endometriosis patients, question 5 (see Table 1) “How much does it bother you in the pain drawing if the body outline is not sex‐adapted?” with three possible responses (“bothers me”; “do not bothers me”; and “no opinion”) were formulated. The responses were collected separately for the female and sexless groups, and Fisher's exact test was employed for analysis, dropping the “no opinion” answers.

#### 2.5.6 Age and importance of identification (Hypothesis 2.6)

In order to detect if there is a statistically significant correlation between the age of the endometriosis patients and the importance of identification with the body outline, question 6 (see Table 1) “Please indicate your age” was formulated and combined with question 4 “When you draw in your endometriosis‐specific pain how important do you think it is that the HBOs is sex‐adapted?” Statistical testing of the hypothesis is performed using a two‐sided Welch *t*‐test for two samples without the variable “no opinion” from question 4.

#### 2.5.7 Age and perceptions of interference with non‐conformity (Hypothesis 2.7)

In order to detect if there is a statistically significant correlation between the age of the endometriosis patients and the disturbing perception of the non‐adaptability of the body outline to the sex, question 6 (see Table 1) “Please indicate your age” was combined with question 5 “How much does it bother you in the pain drawing if the body outline is not sex‐adapted?” Statistical testing of the hypothesis was performed using a two‐sided Welch t‐test for two samples without the variable “no opinion” from question 4.

## 3 RESULTS

Forty‐nine pain drawings and questionnaires were evaluated from participants with an average age of 30.24 years (median 28 years). The age range varied from 16 to 53 years, with the most frequent age group being 20 years old. The majority of participants were between 21 and 30 years old. Twenty‐three sexless and 26 female pain drawings were analyzed. Four participants did not provide their age.

### 3.1 Comparing the differences between the pain extents drawn on female and sexless pain drawings (Hypothesis 1.1)

A heatmap (see Figure 6) generated by the nearest neighbor plugin from Pain2D‐Tool shows log‐10‐scaled RZIs in the upper left quadrant and lower right quadrant, calculated only between PDs within the sexless (blue rows and columns in heatmap) and female groups (red rows and columns), respectively. The lower left and upper right quadrants are reflected at the diagonal. The sub‐squares do not visually reveal any differences in drawing any pain extents within the groups female or sexless or between female and sexless. The null Hypothesis 1.1 that intra‐ and inter‐group RZIs do not differ cannot be rejected. The same RZIs are depicted as boxplots (Figure 5) and contained the following statistics ((f): min–max = [0; 0.444], mean = 0.113, Med = 0.101, *SD* = 0.158; (gn): min–max = [0; 0.440], mean = 0.082, Med = 0.059, *SD* = 0.161; (f‐gn): min–max = [0; 0.362], mean = 0.098, Med = 0.082, *SD* = 0.127) for all three PD pairs (gn stands for comparing sexless (gender‐neutral) PDs to each other; f, comparing female PDs to each other, and f‐gn stands for comparing sexless to female PDs). Again the null hypothesis cannot be rejected. If a significant difference between (f) and (gn) PDs would be seen, then these two boxplots would have higher RZIs which would indicate that the PDs are more similar to each other. In addition, the (f‐gn) boxplot would have to be in the same place, indicating that the two groups are not similar in comparison and are therefore separable from each other. The RZIs of the PD pairs of the sexless to female (f‐gn) would have to be significantly higher than the other pairs which were not the case, indicating that there is no significant difference.

### 3.2 Comparing the pixel count between the pain extents drawn on female and sexless pain profiles (Hypothesis 1.2)

**FIGURE 7:**
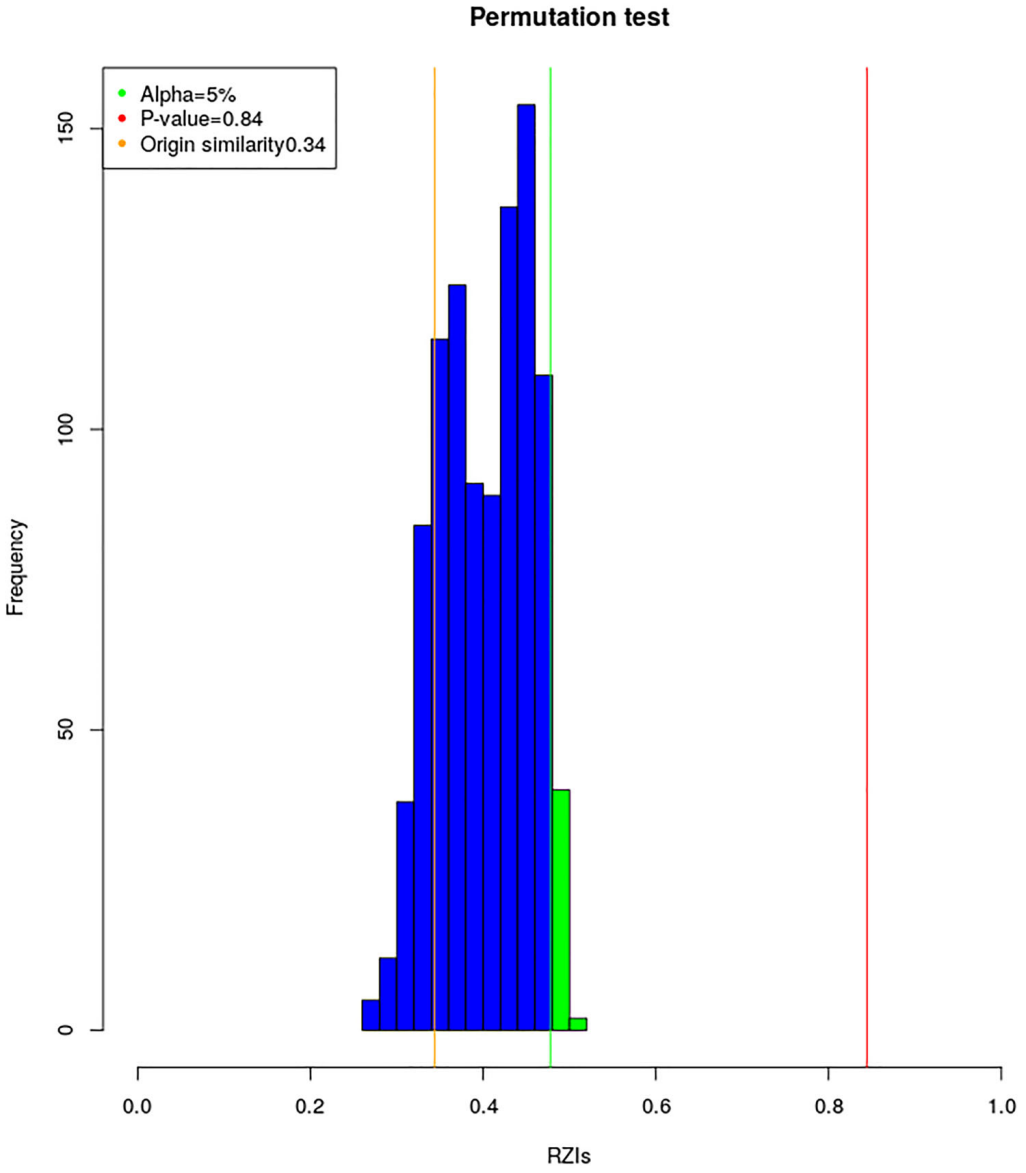
The permutation test histogram is an output of the permutation test plugin from Pain2D‐Tool. It is based on 1000 Ruzika similarity index values for permutation pain profiles, and is used to calculate a *p*‐value and to show where the Ruzika similarity index value of the original data lies in the histogram.

Differences in pain distributions were visually observed between female and sexless patients. The colors in the patterns indicate the proportion of patients who marked the corresponding area. The sexless group marked their pain in more areas (1%–27%) than the female group (1%–13%). Additionally, 65% and 63% of the female and sexless groups, respectively, achieved the largest pixel overlap. A permutation test (see Figure 7) was conducted to determine the significance of the observed differences, using a significance level of alpha = 0.05. The test yielded a high *p*‐value (.86), indicating that the null Hypothesis 1.2 that both groups mark the same extents cannot be rejected.

**FIGURE 8:**
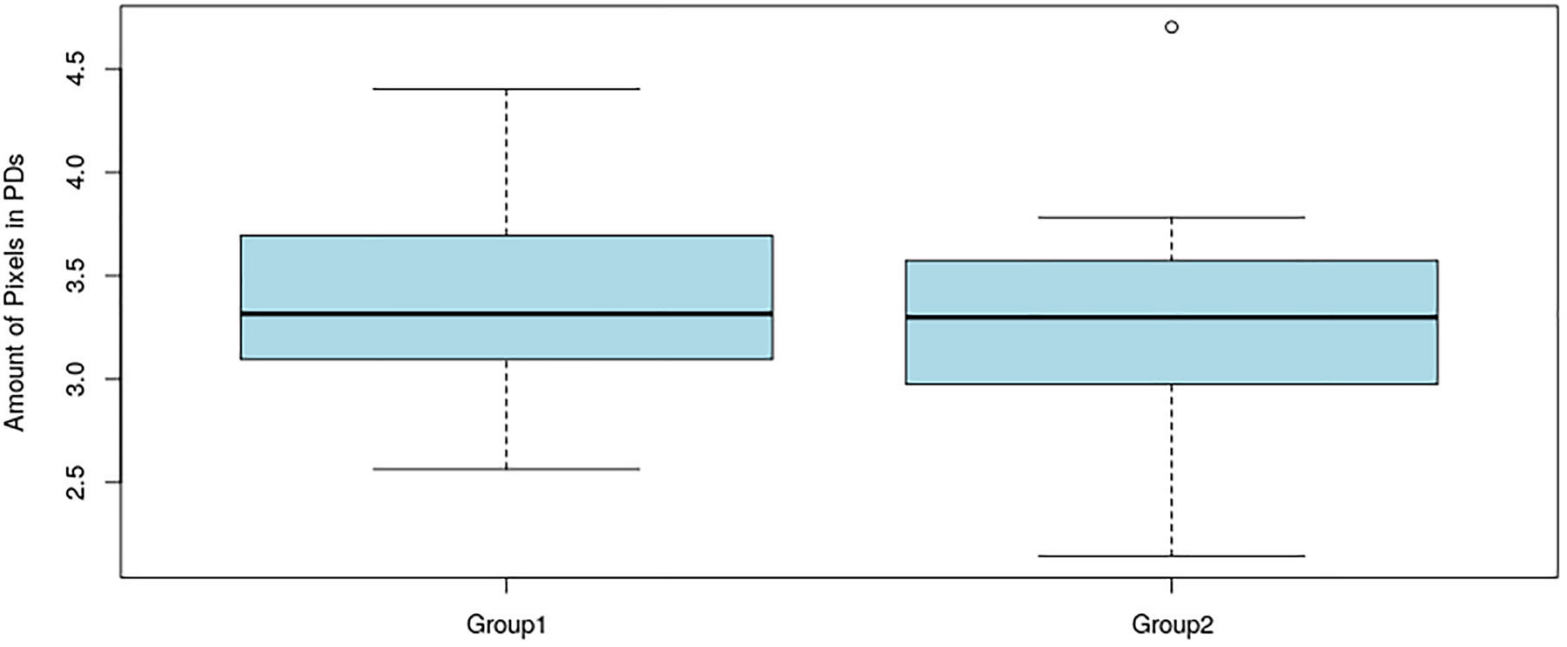
The boxplots are displaying pixel counts which were log10 transformed for all pain drawings in two groups: female (Group1, f) and sexless (Group2, gn) resulting in the following statistics: (f): min–max = [2.564; 4.402], mean = 3.367, Med = 3.316, *SD* = 0.399; (gn): min–max = [2.143; 4.701], mean = 3.274, Med = 3.299, *SD* = 0.500.

Pixels from all PDs for each group: female (Group1) and sexless (Group2) were counted and log10 applied to the values (Figure 8) and resulted in ((f): min–max = [2.564; 4.402]; mean = 3.367; Med = 3.316; *SD* = 0.399; (gn): min–max = [2.143; 4.701]; mean = 3.274; Med = 3.299; *SD* = 0.500). Wilcoxon signed‐rank test: *W* = 347.5, *p* = .4967), *t*‐test: *p* = .474).

### 3.3 Suitability of the HBOs (Hypothesis 2.1)

The related question 1 (see Table 1) whether they found the corresponding HBO suitable was answered by a total of 48 subjects, of which 43 responses (89.58%) were “yes” and 5 responses (10.42%) were “no.” From the group which had sexless PDs, 21 answered with yes, 2 with no. From the group with female PDs, 22 answered with “yes” and 3 with “no.” Fisher's exact test was used and resulted in (*p* = 1; 95% CI: 0.147–18.613; odds ratio: 1.421).

### 3.4 Sex neutrality or femininity of the body outline (Hypothesis 2.2)

Out of a total of 22 people with sexless PDs, 14 people (63.64%) answered “yes” and 8 people (36.36%) answered “no” to the question whether they considered their corresponding HBO sexless. Out of a total of 25 individuals with female PDs, 9 individuals (35%) answered “yes” and 16 individuals (64%) answered “no.” Fisher's exact test did not indicate significant differences in the answers (*p* = .082; odds ratio: 3.033; 95% CI: 0.815–12.151).

### 3.5 Sex recognition characteristics (Hypothesis 2.3)

Question 3 from Table 1 was answered by 36 persons (sexless PDs = 20; female PDs = 16) (for detailed information see Table 2). Chi‐squared test revealed that the null hypothesis cannot be rejected **(*p* = .3964)**.

**TABLE 2:** Collected results from Question 3 (see Table 1).

Feature∖Group	Non‐gender	Female	Sum
Head	6	3	9
Shoulders	5	2	7
Arms	5	0	5
Legs	5	2	7
Buttocks	6	2	8
Abdomen	5	0	5
Waist	10	8	18
Chest	14	12	26
Feet	3	1	4
	59	30	

The most frequently marked feature 26 out of 36 individuals (72.22%) that contributed to sex identification was the breast. Only slight differences were discernible between the groups sexless or female (14:12). The second most frequently selected feature was the waist (18 persons or 50%), with equally small differences between the groups (10:8). All other characteristics were reported more frequently within the group sexless. The characteristics head, shoulders, arms, legs, buttocks and abdomen are similarly represented by 5 and 6 persons, respectively, while the characteristic feet is indicated by only 3 persons. In the female group, the head is indicated by 3 persons as a feature for sex recognition. The remaining characteristics vary between 0 and 2 persons.

For the formulated hypothesis it is valid in the sample that the alternative hypothesis can rather be accepted, and the features here consist of the chest and the waist.

### 3.6 Importance of identification (Hypothesis 2.4)

Out of 48 persons, 25 (52.08%) answered question 4 with (sexless): “important” = 14; “not important” = 9; “no opinion” = 0, (female): “important” = 11; “not important” = 9; and “no opinion” = 5.

The “no opinion” answers were dropped. Fisher's exact test shows did not indicate any strong dependence between the variables (*p* = .763; odds ratio: 0.790; 95% CI: 0.197–3.130).

### 3.7 Perception of disturbance in case of non‐conformity (Hypothesis 2.5)

Out of 48 persons, 22 (45.83%) answered question 5 with (sexless): “bothers me” = 8; “doesn't bother me” = 9; “no opinion” = 6, (female): “bothers me” = 8; “doesn't bother me” = 13; and “no opinion” = 4. The “no opinion” answer was dropped. Fisher's exact test shows again no clear dependency between the variables (*p* = .4501; odds ratio: 0.473; 95% CI: 0.0743–2.683).

### 3.8 Age and importance of identification (Hypothesis 2.6)

Statistical analysis of the Hypothesis 2.6 is performed using a two‐sided Welch *t*‐test for two samples without the “no opinion” answers. The test shows that the null hypothesis cannot be rejected (*p* = .663). The mean values of age are 28.824 in the “unimportant” group and 30.125 in the “important” group. The medians are 26 (“unimportant”) and 29.5 (“important”) years.

### 3.9 Age and perceptions of interference with non‐conformity (Hypothesis 2.7)

The two‐sided Welch *t*‐test shows that the null hypothesis cannot be rejected (*p* = .650). There is no statistically significant association between the age of endometriosis patients and the disturbance perception of the non‐adaptation of the body outline to sex. The mean values of age in the group “bothers me” are 28.8 and in the group “does not bother me” 28.8. “doesn't bother me” group are 30.238. The medians are 28 years in both groups.

## 4 DISCUSSION

Past research has shown that pain drawings often utilize HBOs that are not sex specific, which can be problematic (Alonso‐Blanco et al., 2012; Türp et al., 1998). As a result, it is essential to consider sex and diversity when using pain drawings for pain localization. This study found that there were no significant differences in the drawing of endometriosis‐specific pain between pain drawings containing female or sexless HBOs. In addition, there were no significant differences in the location or number of pixels of pain extents drawn by female patients. Our findings support previous research by Egsgaard et al. (2016) which also found no significant differences in the drawing of pain extents in pain drawings that contained female and male HBOs, and were drawn by female participants. However, pain profiles did reveal that different areas were drawn on the sexless body outline, such as more pain drawn in the legs, compared to the female body outline. This difference may be due to the small sample size and the resulting data noise, which was confirmed by the calculated RZIs that showed small differences. Several participants were unable to recognize the sex neutrality of the HBOs developed in the study. Compared to the female group, the group with sexless HBOs identified more features as important. The chest and hips were found to be significant for sex recognition and should be given special attention when designing sexless HBOs. For the arms and abdomen, 5% of participants from the sexless group and none from the female group indicated that these features should be used to identify sex. The situation was similar for the buttocks, with six subjects from the sexless group and two from the female group selecting this feature. This disparity could be attributed to the fact that each group was only shown their own HBOs, and the female group tended to focus more on unique sex characteristics, particularly the breasts. Additionally, there may have been a bias due to the wording of the questionnaire, where the associated question with the words “If yes” referred to the previous question, resulting in fewer participants with female HBOs responding to this question. Furthermore, some participants reported feeling uncomfortable when the body outline used in pain drawings did not match their sex.

The study also explored the subjective meaning of diversity and found that endometriosis patients tend to consider the body outline, in any case, as appropriate to draw perceived endometriosis‐specific pain. However, approximately 10% of participants indicated that they did not consider the body outline presented to be suitable for this purpose, regardless of whether they were presented with a sexless or female body outline. The appropriateness of HBOs is an important factor in allowing people to clearly communicate their pain.

The investigation aimed to determine if the HBOs were sexless based on questionnaires. Unfortunately, the results were inconclusive. However, there is some evidence suggesting that sexless HBOs are preferred over female HBOs. It is worth noting that some survey questions may have produced response effects. For example, some participants tended to select the neutral answer option when they were unsure or insecure, a phenomenon known as “tendency towards the middle,” as described by Bogner and Landrock (2015). While this may have caused some limitations, it can also prevent systematic bias. Menold and Bogner (2015) suggest that having a neutral category prevents participants from feeling pressured to choose a positive or negative answer, thereby avoiding biased responses.

No correlation was found between the age of endometriosis patients and their level of identification with the HBOs or the degree to which non‐conformity of the body outline was disturbing. Interestingly, older individuals in the sample expressed a greater importance in identification with the body outline, but were less bothered by non‐conformity, while younger individuals were more disturbed by non‐conformity but placed less importance on identification. However, it should be noted that this study focused on endometriosis patients, who are all biologically female. Nevertheless, the results highlight the importance of diversity and adaptation of the body schema to one's sex in pain drawings. While it may not be a priority for some individuals, it is important to others. Therefore, it is necessary to address the needs and requirements of all individuals to ensure that everyone can accurately represent their pain in pain drawings. Further studies should also consider intersex and transgender individuals, as well as those who do not conform to normative gender groups. The development of more inclusive HBOs is also of great importance.

A follow‐up study with a larger sample size would be beneficial to validate the current results, as the observed trends may be influenced by the limited number of cases or the specific endometriosis condition of the female participants. Additionally, investigating sex differences in pain localization using male, female, and sexless HBOs could yield interesting findings. It is plausible that discrepancies exist between the frequently used male pain drawing HBOs and those of the opposite sex. Since the current study only focused on women, future research should encompass men and non‐binary individuals to explore potential gender variances.

The female TPD did not indicate the spina iliaca anterior that some of the participants might have expected as another anatomical landmark. This could have a small bias on the results of the study reducing the differences between female and sexless HBOs.

## 5 CONCLUSIONS

The results of this study suggest that the sex of the HBOs used in pain drawings has no or little effect on the pain area, contradicting the findings of a 2016 study by Egsgaard et al. No statistically significant differences were detected in pain localization or the area of pain between the female and sexless HBOs used in this study. However, it was noted that the body outline used could still be improved, as it was not perceived as suitable by all participants. This highlights the importance of paying attention to the sex of HBOs in pain drawings, as it can impact the identification and comfort of patients. Developing more adequate HBOs, focusing on features such as the waist and breasts, could be a crucial step toward improving pain localization using pain drawings. If a larger study with a larger sample were to confirm the lack of differences in pain localization between sexless and sex‐specific HBOs, a sexless body outline could potentially be used universally for pain drawings for all patients, rather than the male body outline commonly used in practice. Overall, this study emphasizes the need for healthcare practitioners to consider the impact of sex on pain experiences and to prioritize inclusive and appropriate methods for pain assessment and communication.

## AUTHOR CONTRIBUTIONS

Conceptualization: Frank Klawonn and Natasza Szczypien; Methodology: Frank Klawonn and Natasza Szczypien; Software: Natasza Szczypien; Formal analysis: Frank Klawonn and Natasza Szczypien; Medical data investigation: Zino Ruchay; Writing—original draft preparation: Natasza Szczypien and Zino Ruchay; Writing—review and editing: Natasza Szczypien and Zino Ruchay; Visualization: Natasza Szczypien; Supervision: Frank Klawonn, Sandra Verena Müller, and Claudia Kaiser; Project administration: Frank Klawonn and Natasza Szczypien.

## CONFLICT OF INTEREST STATEMENT

This manuscript has been submitted as original research. The authors certify that they have no affiliations with or involvement in any organization or entity with any financial interest or non‐financial interest in the subject matter or materials discussed in this manuscript.

### PEER REVIEW

The peer review history for this article is available at https://publons.com/publon/10.1002/brb3.3285

## What's known/what's new statements

•Traditional human body outlines (HBOs) used for pain drawings are often based on male anatomy, which may not accurately represent female or gender‐diverse patients' experiences. Women with chronic musculoskeletal pain tend to draw larger pain areas than men, and may require more detailed HBOs to accurately depict their pain.

•In our study, sexless and female HBOs were used in pain drawings for endometriosis and had no effect on pain area, and no statistically significant differences were found in pain localization or area between female and sexless outlines.

## Supporting information

Supporting Information

## Data Availability

The data that support the findings of this study are available on request from the corresponding author. The data are not publicly available due to privacy or ethical restrictions.
