# Supplementary material for: Sex‐specific differences in pain localization in female patients with endometriosis: A comparison of sexless and female human body outlines
Source: Brain Behav. 2023 Oct 18;13(12):e3285. doi: 10.1002/brb3.3285 (PMC10726775; doi:10.1002/brb3.3285)
Supplement: Supplementary file 1 — Supporting Information [file BRB3-13-e3285-s001.pdf]

Ostfalia
Hochschule für angewandte Wissenschaften

Studie

zum Schmerzzeichnungsprogramm „Pain2D“ für die Weiterentwicklung von medizinischen Diagnosetools

Sehr geehrte Interessentinnen,

um zukünftige medizinische Diagnosetools weiterzuentwickeln, untersuchen wir anhand einer wissenschaftlichen Studie das Schmerzzeichnungsprogramm „Pain2D“. Die Teilnahme ist freiwillig und anonym. Weitere Informationen finden Sie auf dem Informationsblatt.

Wir freuen uns über Ihre Teilnahme!

Markieren Sie bitte auf der nächsten Seite Ihre **Endometriose-spezifischen Schmerzen**, welche Sie im Unterleib und/oder im Rückenbereich empfinden.
Befolgen Sie dazu bitte die folgenden Zeichnungskriterien:

1. Die Schmerzen dürfen ausschließlich mit einem **schwarzen Filzstift** gezeichnet werden.
2. Bitte malen Sie alle Schmerzpunkte aus, die Sie empfinden (siehe Figur links).
3. Kreuzchen, unausgefüllte Punkte, Pfeile usw. sind nicht erlaubt (siehe Figur rechts).

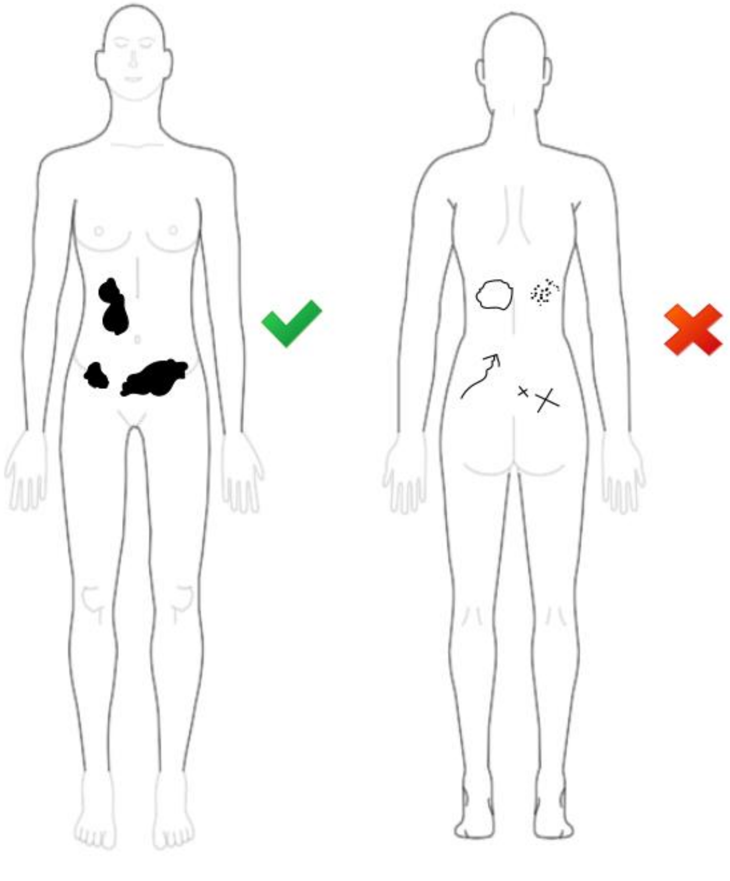

Bitte zeichnen Sie jetzt Ihre Schmerzen auf der nächsten Seite!

[redacted]

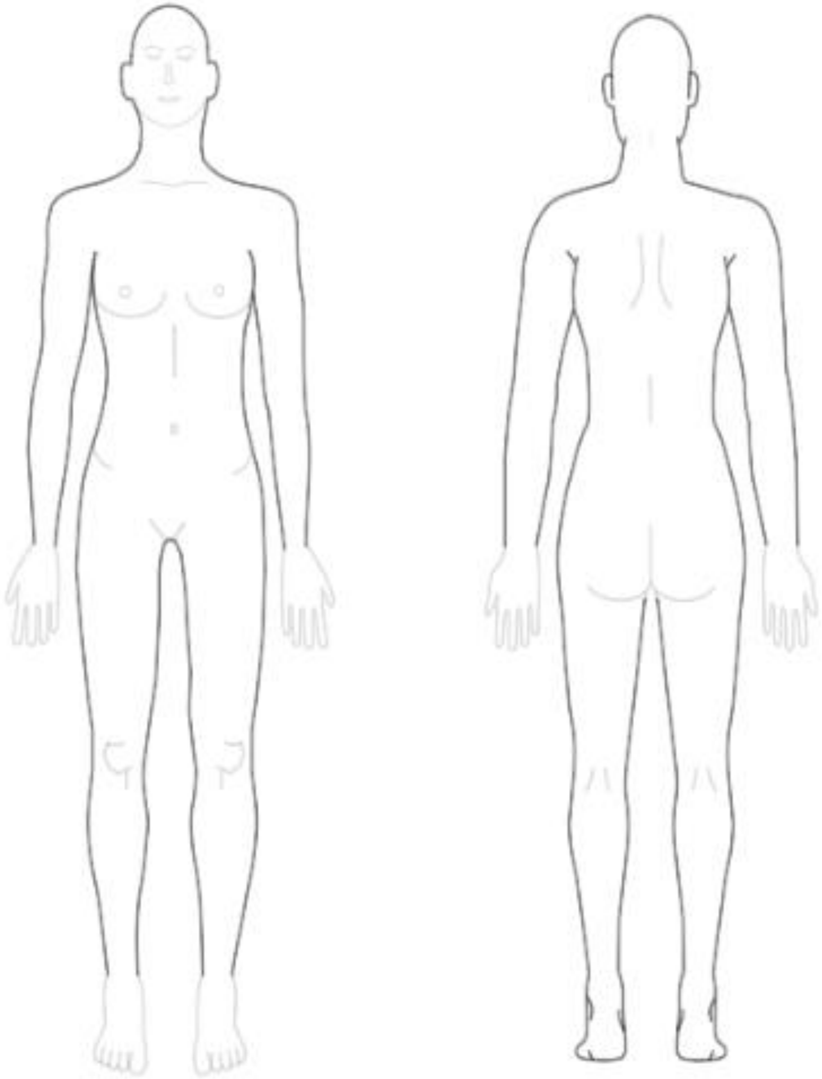

[redacted]

[redacted]

Ostfalia
Hochschule für angewandte
Wissenschaften

1. Können Sie sich mit der Körpervorlage identifizieren?
   - ❑ ja
   - ❑ nein

2. Halten Sie die Körpervorlage für geschlechtsneutral?
   - ❑ ja
   - ❑ nein

3. Falls ja: Anhand welcher Merkmale sehen Sie, ob die Körpervorlage geschlechtslos bzw. weiblich ist?
   - ❑ Kopf
   - ❑ Schultern
   - ❑ Arme
   - ❑ Beine
   - ❑ Gesäß
   - ❑ Bauch
   - ❑ Taille
   - ❑ Brust
   - ❑ Füße

4. Wenn Sie Ihre Endometriose-spezifischen Schmerzen einzeichnen, für wie wichtig halten Sie es, dass die Körpervorlage Ihrem Geschlecht angepasst ist?
   - ❑ wichtig
   - ❑ keine Meinung
   - ❑ unwichtig

5. Wie sehr stört es Sie bei der Schmerzzeichnung, wenn die Körpervorlage nicht Ihrem Geschlecht angepasst ist?
   - ❑ stört gar nicht
   - ❑ keine Meinung
   - ❑ stört mich

Patientencodierung (bitte frei lassen)

❑ A
❑ F

Pat_nr		
0	❑	❑
1	❑	❑
2	❑	❑
3	❑	❑
4	❑	❑
5	❑	❑
6	❑	❑
7	❑	❑
8	❑	❑
9	❑	❑

Informationsblatt zum Mitnehmen

Sehr geehrte Teilnehmerinnen,

im Rahmen des Masterstudiengangs Präventive Soziale Arbeit an der Hochschule für Wissenschaften Ostfalia in Braunschweig/Wolfenbüttel untersuchen wir das Schmerzzeichnungsprogramm „Pain2D“, welches entwickelt wurde, um zukünftig medizinische Diagnosen anhand von Schmerzzeichnungen zu erleichtern. Als Teilnehmerin leisten Sie einen wichtigen Beitrag für die Weiterentwicklung neuer, zukünftiger Diagnosetools mit Schmerzzeichnungen für Ärzt*innen aus aller Welt. Die Studie ist vollständig **anonym** und es wird eine strenge Beachtung des Datenschutzes gewährleistet. Die Daten werden nicht an Dritte weitergegeben und nach Abschluss der Studie aus unserer Datenbank vollständig gelöscht. Die Teilnahme ist **freiwillig** und entstehen keinerlei Kosten. Bei Fragen rund um die Studie können sie sich gerne per E-Mail an uns wenden:

na.szczypien@ostfalia.de
z.ruchay@ostfalia.de

Vielen Dank für Ihre Teilnahme!
